# Change in Objective Measure of Empathic Accuracy Following Social Cognitive Training

**DOI:** 10.3389/fpsyt.2019.00894

**Published:** 2019-12-10

**Authors:** Kristen M. Haut, David Dodell-Feder, Erin Guty, Mor Nahum, Christine I. Hooker

**Affiliations:** ^1^Department of Psychiatry & Behavioral Sciences, Rush University Medical Center, Chicago, IL, United States; ^2^Department of Psychology, University of Rochester, Rochester, NY, United States; ^3^Department of Psychology, Pennsylvania State University, State College, PA, United States; ^4^School of Occupational Therapy, Faculty of Medicine, Hebrew University, Jerusalem, Israel

**Keywords:** empathy, cognitive training, empathic accuracy, social cognition, social cognitive training

## Abstract

**Background:** The capacity for empathy plays an important role in interpersonal relationships and social functioning, and impairments in empathy can have negative effects on social interactions and overall social adjustment. This suggests that empathy may be a critical target for intervention in individuals who struggle with social interactions, yet it is unclear if the skills required for empathy are malleable. This study investigates the efficacy of targeted social cognitive training for improving empathic skills.

**Methods:** Forty-five individuals (mean age = 24) were included in this study. Twenty-four individuals were allocated to the active social cognition training group and 21 individuals were allocated to a computer games control condition. Subjects completed approximately 10.5 h of training over two weeks. Pre- and post- training, they completed measures of empathy and emotion recognition, including the Interpersonal Reactivity Inventory (IRI) and an empathic accuracy task. ANOVA and regression analyses tested changes in participants’ performance on the empathic accuracy task and scores on the IRI subscales were used to assess the effect of the social cognitive training.

**Results:** Repeated measures ANOVA show that there is a significant group by timepoint interaction on the Empathic Accuracy task, with individuals who completed the social cognition training showing a significant improvement in performance following training. There were no significant changes for either group on any of the self-report IRI subscales. Individuals in the active training group show significant improvement on negative valence videos and a trend towards improvement on positive valence videos. In addition, individuals in social cognition active training group who reported higher intrinsic motivation demonstrated greater improvement on the Empathic Accuracy task.

**Conclusions:** Individuals who completed a computerized social cognition training program demonstrated improved performance on a rater objective measure of empathic accuracy while individuals who completed a computer game control condition did not demonstrate any significant changes in their performance on the empathic accuracy task. These results suggest that targeted training in social cognition may increase empathic abilities, even in healthy individuals, and that this training may be beneficial to individuals with social cognitive deficits.

## Introduction

Empathy is the ability to perceive, understand, and share others subjective emotional thoughts or experiences, as well as to generate appropriate responses to others affective states. This capacity for feeling and understanding the experience of another individual plays an important role in the development and maintenance of close personal relationships (1) and prosocial behaviors (2). Empathy is critical for healthy social functioning in central relationships, such as between spouses (3). Impairments or deficits in empathy can have negative effects on key social interactions and the ability to provide social support (4). On the other hand, higher levels of empathy have been associated with better overall adjustment and fewer emotional problems in adolescents with poor peer relationships (5). The importance of empathy in developing and maintaining social relationships suggests that it may be a critical target for intervention in individuals who struggle with social problems and/or appropriate empathic functioning. However, it is unclear which skills that contribute to an individual’s empathic functioning are malleable, and if so, to what extent those skills can change or improve in response to targeted treatment. The purpose of the present study is to assess the capability of a targeted social cognitive training (SCT) paradigm to improve empathic skills as measured by both a rater objective empathic accuracy task and a subjective empathic reactivity self-report measure.

Empathy is a complex domain with multiple component processes, which may be differentially responsive to training. One primary distinction is between cognitive components of empathy, such as the recognition, identification and representation of the internal affective states of another, and emotional components of empathy, which refers to an individual’s internally-generated affective response to the other person, allowing for experience-sharing (6). Cognitive components of empathy are particularly influenced by general cognitive skills, such as speed of processing, that are vulnerable to deterioration with age or psychiatric illness (7). Cognitive empathy requires the individual to accurately detect and identify others’ emotional experiences as well as engage in perspective taking and theory of mind (8) and this component of cognitive empathy may be particularly responsive to behavioral interventions. Interventions such as the Micro-Expression Training Tool (9) have been developed to aid individuals in being better able to recognize and interpret subtle facial indications of affective expressions. Social cognition training has also been shown to produce a moderate effect size improvement in elements necessary for cognitive empathy such as facial affect recognition and theory of mind in individuals with schizophrenia (10). This research suggests that cognitive skills required for empathic abilities may respond to direct behavioral interventions.

Empathy and composite social cognitive skills such as affect recognition, perspective taking, and theory of mind are associated with individual differences in personality (11), age (7), motivation (12), and general cognitive abilities (13). Deficits in empathy have also been associated with impaired social functioning or impaired interpersonal interactions in a number of psychological problems and disorders. For example, there is increasing evidence that children with disruptive behavior disorders such as Conduct Disorder and Oppositional Defiant Disorder show deficits in emotion recognition, a skill necessary to effectively mentalize about another’s experience and engage empathic responses (14, 15). Furthermore, children with these disorders show deficits in emotional experience sharing, a component of affective empathy (16), and these deficits are maintained in adults with psychopathy (6). Deficits in social-cognitive skills required for empathic abilities are also present in autism (6) and in schizophrenia (13). There is evidence that impairments in empathy within these groups have a significant influence on the individual’s social and community function, beyond the effects of general cognition or symptoms (17, 18). Empathy is partially heritable (19) and variance in empathic performance has been linked to polymorphisms of the oxytocin receptor gene (20–22). However, developmental effects on empathy are profound in early childhood and adolescence (23, 24) and are dependent on environmental and experiential factors like family environment, maternal synchrony and mimicry (25–27). Taken together, these factors suggest that empathy may be a critical target for developing interventions aimed at improving social functioning and interpersonal relationships.

Detecting changes in empathy following behavioral interventions requires the use of reliable measures capable of quantifying the different skills required for accurate and successful empathy. Empathy is often measured by self-report measures such as the Interpersonal Reactivity Index (IRI), which broadly assesses an individuals’ views of their own empathic ability (28). This measure evaluates individuals on multiple components of dispositional empathy that encompass both cognitive and affective empathy, including perspective taking (the ability to view a situation from another’s perspective), empathic concern (feeling compassion towards another person), personal distress (feeling distress in response to seeing other’s distress) and fantasy (the ability to imagine oneself in another situation). The IRI has been used extensively in the study of empathy including as a measure of change in empathy over time in adolescents (29), impaired empathy associated with psychosis in individuals with borderline personality disorder (30), and identifying neural correlates of empathy (31, 32). The four subscales individually address different components that make up empathy more broadly and this literature demonstrates the utility of the IRI as a measure of self-reported empathy. However, the empathic traits measured by the IRI are based on subjective reporting and do not always correlate strongly with performance measures of empathic abilities or may not clearly reflect an individual’s exhibition of empathy in naturalistic settings (33, 34).

The limitations of self-report measures of empathy like the IRI demonstrate the necessity of also assessing empathy using more objective measures when evaluating behavioral change in the context of social cognitive training. One skill especially important to empathy that researchers have developed an objective measure for is of empathic accuracy; specifically, how correctly the perceiver is able to infer the thoughts and/or feelings of another person in everyday interactions or simulations of those interactions (35). Empathic accuracy can be measured using a paradigm that includes a video-cued procedure comparing the perceiver’s affective ratings of a social target with that target’s self-report. Moment-to-moment agreement between the perceiver and target can then be used to generate an objective empirical accuracy measure for the perceiver. These paradigms require the perceiver to use facial expressions, verbal content, vocal tone, posture, and other non-verbal cues to continuously track changes in emotional intensity, which closely mimics the skills needed in real-life social situations and requires coordination of abilities particularly tied to cognitive empathy (36, 37). Empirical empathic accuracy measures are an important addition to empathy research as they have been shown to be reliable, can be applied in a variety of contexts, do not correlate particularly strongly with self-report measures and are more predictive of life outcome measures (38). These tasks are sensitive to inter-personal differences in empathic ability (39) and difficulty in empathic accuracy within a couple is linked to interpersonal aggression (4). In addition, this task is sensitive to performance changes with age, especially in accurately identifying the expression of negative affect (40) as well as performance deficits in individuals with psychiatric disorders (41). Individual performance on the empathic accuracy paradigm has also been shown to change with pharmacological manipulation such as oxytocin administration (42) which suggests it may be a measure particularly sensitive to interventions such as social cognitive training.

The social cognitive training tested in this study (Targeted Social Cognitive Training, SCT) utilizes a plasticity-based training approach that aims to strengthen the underlying components of cognitive processing to effect behavioral change. This approach differs from some traditional intervention approaches for social skills that are more group process oriented and focus on providing strategies for coping with social situations (43). Instead, targeted cognitive training uses adaptive, individualized exercises to enhance brain plasticity in brain networks that process socially-relevant stimuli (44). In the SCT, the skills trained include many required for accurate cognitive empathy including face and affect perception in a variety of contexts, prosody and theory of mind (45). Importantly, the empathic accuracy task used to test objective cognitive empathy performance in this study requires similar underlying skills; however, this particular task was not included in the training paradigm and thus requires the transfer of improvements to a novel task.

This study tests whether a targeted social cognitive training protocol improves both objectively measured empathic accuracy and self-reported empathy (via the IRI) in healthy young adults. The computerized SCT was developed initially to address the primary social cognitive domains known to be deficient in individuals with schizophrenia, including affect perception, social cue perception and empathy (46). The goal of this study is to evaluate whether training using the SCT will improve performance on untrained behavioral measurements of empathy in healthy controls. We hypothesize that individuals who received SCT would demonstrate improved performance on an empathic accuracy measure that requires use of these skills while individuals in a computer games control condition would not. In addition, we hypothesized that the objective empathic accuracy measure would be a more sensitive measure of training-based change than subjective empathy measures following completion of the treatment protocol. In addition, SCT relies on active engagement in the training tasks in order to induce neuroplasticity and thus may also be susceptible to variations in motivation (47, 48). In order to test this, participants completed the Intrinsic Motivation Inventory (49), a self-report measure of treatment engagement and perceived value, to allow for assessing the moderating effects of motivation on SCT efficacy.

## Materials and Methods

### Participants

This study was approved by the Harvard Institutional Review Board (IRB) and all participants gave written informed consent after receiving a description of the study. Individuals ages 18 to 30 were recruited from the local community to participate in a study. Individuals were excluded for: 1) lifetime history of an Axis I Disorder or substance abuse/dependence in the last 5 months based on the SCID (50); 2) history of major medical illness, neurological problems or loss of consciousness >30 min due to head trauma; 3) IQ < 70 as assessed by the NAART (51); 4) insufficient fluency or reading ability in English to understand study procedures or complete the training program; or 4) MRI incompatible implants or claustrophobia.

A total of 81 healthy individuals were screened and 9 individuals were excluded. In addition, 10 participants withdrew prior to beginning the training program and 6 were non-compliant with training or withdrew before study completion. Furthermore, pre-test or post-test data on the empathic accuracy task were missing for 4 individuals in the active condition and 7 individuals in the control condition. The final sample included in this study consisted of N = 24 individuals in the active condition and N = 21 individuals in the control condition (**Supplementary Figure 1**, **Supplementary Table 1**). There were no significant differences between groups on age, gender, education or IQ as estimated by the NAART. See demographics reported on **Table 1** for details.

**Table 1:** Sample characteristics and reported motivation.

	Active N = 24	Control N = 21	Group difference	
			Statistic	*p*-value
% Male	58.3%	47%	X^2^ = 0.52	0.47
Mean age (SD)	24.54 (2.92)	24.57 (2.93)	t = ‑0.03	0.97
Mean years of education (SD)	16.29 (1.97)	16.05 (1.56)	t = 0.46	0.65
NAART IQ (SD)	120.46 (6.53)	121.43 (4.91)	t = ‑0.57	0.57
Intrinsic motivation inventory (SD)	170.09 (20.42)	163.37 (22.92)	t = 1.03	0.31
Enjoyment	4.17 (.85)	4.59 (.98)	t = -1.51	0.14
Effort	5.52 (1.12)	5.26 (1.11)	t = .78	0.44
Choice	5.70 (.59)	5.38 (.66)	t = 1.66	0.10
Value	5.04 (1.07)	4.46 (1.18)	t = 1.72	0.09

Demographic characteristics of individuals allocated to the Active and the Control group conditions show no significant differences between groups. The Intrinsic Motivation Inventory was given to subjects post-training and show now significant differences in perceived enjoyment or effort between groups and a trend towards higher choice and value in the individuals who received the Active social cognition training.

### Study Protocol

The present study is part of a larger project investigating behavioral and neural effects of social cognition training. Recruited participants first completed a clinical screening assessment to assure that they met study inclusion criteria. Participants completed a battery of behavioral and neuroimaging measures before and after training. Post-training assessment was scheduled as soon as possible following the completion of the training, typically within a week of the last training session.

Participants were allocated to one of two training groups: the social cognitive training (SCT) group or computer game control (CON) group in a modified “block” randomization procedure, such that the first block of 20 subjects was assigned to SCT and the second block of 20 subjects to the CON condition. After that subjects were alternately assigned—i.e. randomized—in pairs or in 1-to-1 fashion accounting for age and gender to SCT or CON for rest of study. As this was the first application of this social cognition training protocol, this design was used in order to assure social cognition training program was working as designed. In addition, as individuals completed the training at a time and at a location of their choosing, t. The initial allocation of a block of subjects to SCT allowed us to verify specific characteristics of SCT (e.g. length of SCT exercises) in the participant’s “real-world” settings and, thus, ensure that the CON condition matched SCT appropriately. Group assignment was double-blind, in that experimenters who tested participants post-training were blind to the participant’s condition. Participants were told that they would be doing “brain training games” and could receive one of multiple different programs but were not informed of treatment condition or study hypotheses prior to completion of the training and post-training assessment.

### Study Interventions: Targeted Social Cognitive Training (SCT) and Computer Game Control (CON)

Individuals assigned to the SCT condition completed a social cognition training program developed by the Posit Science Corporation and that is detailed elsewhere (46). The program consists of a suite of exercises to train facial emotion recognition, emotional prosody, perspective taking, navigating social interactions and theory of mind (see **Supplemental Table 2** for a brief description of each exercise included in the intervention). Each exercise is adapted in difficulty to individual performance, both during the exercise and between the exercises. Individuals receive feedback on a trial-by-trial basis and also receive feedback on their overall performance after completing each task. Individuals assigned to the CON condition completed a suite of common computer games, such as solitaire and word search, that was accessed and delivered *via* the same website and presentation format as the SCT condition. This active control was chosen to equate interaction with study staff, time on computer and maintain blindness of both participants and study staff.

Training was completed online using brainhq.com and could be monitored by a lab member, remotely as the program records when individuals train as well as their performance data. Participants were invited in for a “training orientation” where a lab member introduced the participants to the training software and participants completed an hour session in lab. Then participants were asked to train for one to three sessions per day (5 days a week) for a total of 15 sessions, each session typically consisting of four training exercises and lasting for 45 min. Overall, individuals received approximately 10.5 h of training over two to three weeks and received financial compensation for each training session completed as well as a bonus if they completed the training within 2 weeks and without skipping more than two days in a row. If participant missed two days of training, then they would receive a communication from the lab monitor as a reminder to continue training. Subjects who did not complete the training also did not attend a post-test assessment and so are excluded from this study.

### Assessments

The full assessment battery consisted of behavioral and neural measures designed to investigate different scientific questions. To address the goals of the current study, the following measures (described below) were included in the assessment battery and performance was analyzed to test a priori hypotheses.

#### Empathic Accuracy Task

In the empathic accuracy paradigm, participants watch a series of short videos in which an individual (i.e. community member) describes a significant life event, and, while viewing each video, the participant is asked to make moment-by-moment inferences about the naturalistically occurring thoughts and feelings of the target individual. More specifically, the participant rates the target individual’s emotional state throughout the videotaped recollection and these ratings are scored for accuracy against the target individual’s *true* thoughts and feelings that were self-reported when the video was originally made. Ratings were made on a 9-point sliding scale that varies from extremely negative to extremely positive. In the task used here, participants watched six videos, 3 positive and 3 negative ones, each lasting for 2–2.5 min long, and provided continuous ratings of how they perceived the target to be feeling at each moment of the video on the same 9-point sliding scale. Participants were instructed to adjust their rating any time they sensed a change in the target’s emotion state. Videos were presented in the same order for each subject and subjects were shown the same videos pre- and post-training. The specific empathic accuracy task and stimuli used in the present research has been used in previous studies (41) and was adapted from a previously developed empathic accuracy paradigm (37).

Participants’ continuous affective ratings were converted into a time series using the average rating for each 2-second epoch for each video. To calculate empathic accuracy, participants’ continuous ratings across these 2-second epochs were correlated with the target’s own continuous ratings across the same epochs for each video. The resulting correlation coefficient (r) between the two-time series is the measure of empathic accuracy, producing a single EA score (correlation) for each video for each individual. Before conducting any statistical analyses, the individual correlation coefficients for both tasks were converted into z scores using Fisher’s technique.

#### Interpersonal Reactivity Index (IRI)

Subjective trait empathy was assessed using the well-validated Interpersonal Reactivity Index (IRI) that measures self-reported dispositional empathy (28). The IRI asks individuals to report how empathic they believe they are on a series of 28-items using a 5-point scale ranging from “Does not describe me well” to “Describes me very well”. The measure is comprised of four subscales that represent distinct components of overall empathy, including empathic concern, perspective-taking, fantasy, and personal distress.

#### Intrinsic Motivation Inventory (IMI)

Following training, participants completed a version of the Intrinsic Motivation Inventory (IMI) (52) which was developed for use in cognitive remediation studies of psychiatric populations (49) to assess each individual’s response and engagement in the training exercises. This scale is a 21-item measure using a 7-point scale ranging from “Not at all true for me” to “Very true for me”. The measure assesses participants’ interest/enjoyment during the training as well as the perceived effort, pressure, choice, and usefulness of the training with questions such as “I thought this activity was quite enjoyable” and “I think this is important to do”. Higher IMI scores suggests greater motivation and engagement in the training and post-training IMI scores were added to the statistical models to determine if intrinsic motivation was related to training effects or overall performance on the empathic accuracy task. The IMI was administered only at post-test as it asks participants to reflect on their training experience in order to assess the individual’s subjective engagement and perceived benefit of completing the training exercises.

### Statistical Analyses

All analyses were performed in R (53). Regression analyses and t-tests were used to assess the effect of demographic characteristics on baseline task performance. Where appropriate, all reported statistical tests were conducted with and without covariation from demographic variables and values reported are without demographic covariation (unless otherwise noted). A repeated measures ANOVA was conducted to assess for group differences and change following training on each of the four subscales of the IRI. In addition to an overall EA score, separate EA scores were computed for the 3 positively-valenced videos and the 3 negatively-valenced ones, as accuracy for negative moods is sometimes found to be greater that accuracy for positive moods (54). The empathic accuracy summary score ranged from an average correlation of 0.529 to an average correlation of 0.775 across all study participants. To compare group difference on the empathic accuracy task, z-scores were averaged across all videos (total EA score) and for positive and negative valence videos separately. A repeated measures ANOVA was conducted to assess for group differences in empathic accuracy performance change following training as well as within-group paired t-tests to determine the pattern of significant results. An additional repeated measures ANOVA with both timepoint and valence included as within-subject variables and training group as a between subject factor was also conducted in order to assess for the effect of emotional valence on training effects. Within and between-group t-tests were performed to explore significant effects. To confirm results, a hierarchical linear model was used to assess the effect of training group on performance. Later stages of the model also use age, gender, NART and overall IMI score as covariates.

## Results

### Participant Characteristics

There were no demographic differences between the groups on gender, age, education or IQ as estimated by the NAART (**Table 1**). There was no significant effect of these demographic variables on baseline empathic accuracy performance across groups; however, higher levels of education were associated with higher empathic accuracy scores at a trend level of significance [t(44) = 1.89, p = 0.07, r = 0.28]. There were no demographic effects on baseline IRI ratings or on any IMI scores post-training. All subjects included in the analyses completed the assigned training (10.5 h). Subjects completed the training in 5 to 15 days, with an average completion time of 11.43 days (sd = 2.64). Length of time to complete the training was not found to be a significant predictor of change in task performance.

### Objective Empathy—Empathic Accuracy Task

**Figure 1:**
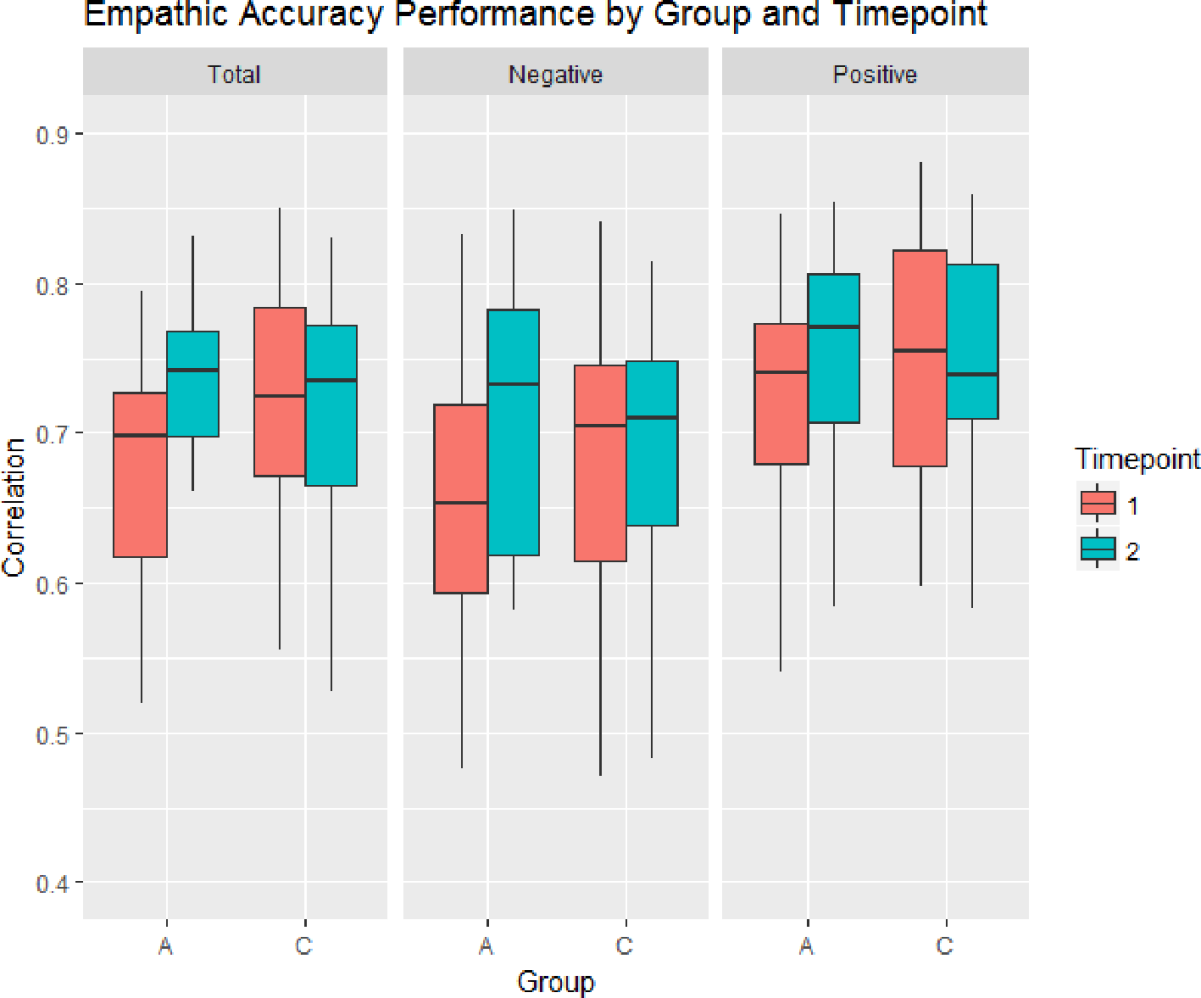
Change in the Empathic Accuracy correlation score for individuals who received the Active social cognition training condition (A) and the Control computer games condition (C). Individual in the Active group showed significant changes in overall empathic accuracy performance from pre-test to post-test. Increased performance was found for videos with both positive and negative valence.

There were no significant differences at baseline between the active SCT group and the CON group for overall empathic accuracy performance (F(1,43) = 2.43, p = 0.13, η^2^_G_ = 0.04) and no significant differences between groups at followup (F(1,43) = 0.25, p = 0.62, η^2^_G_ = 0.001). However, repeated measures ANOVA shows that there was a significant group (SCT/control) by time point (pre/post training) interaction (F(1,43) = 4.254, p = 0.045, η^2^_G_ = 0.02). Within-group paired t-tests indicate that the SCT showed a significant increase in overall empathic accuracy score following training [t(23) = 2.58, p = 0.008, *d* = 0.53] whereas CON did not [**Figure 1**; t(20) = ‑0.559, p = 0.582, *d* = 0.12].

Across time points, both groups had higher empathic accuracy scores on positive valence videos than negative valence video [F(1,43) = 11.78, p = 0.002, η^2^_G_ = 0.06] and there were no group differences on scores for positive or negative valence videos [F(1,43) = 0.02, p = 0.889, η^2^_G_ = 0.001]. There was also no differential effect of valence on the change revealed by the group by time point interaction [F(1,43) = 0.140, p = 0.710, η^2^_G_ < 0.001] suggesting that the significant improvement in empathic accuracy performance in the SCT does not differ for positive valence videos or negative valence videos. Individuals in the SCT group demonstrated a significant improvement on negative valence videos [t(23) = 2.497, p = 0.01, *d* = 0.51] and an improvement on positive valence videos [t(23) = 1.918, p = 0.03, *d* = 0.39], whereas there was no change in the CON group on either negative [t(20) = ‑0.350, p = 0.730, *d* = 0.08] or positive [t(20) = ‑0.551, p = 0.588, *d* = 0.12] videos. See **Table 2** for the empathic accuracy correlations for each group and valence at each time point as well as between group t-test comparisons.

**Table 2:** Empathic accuracy performance.

	Active N = 24	Control N = 21	Group difference		
			Statistic	*p*-value	Effect size (d)
**Pre-training empathic accuracy score**					
Total	0.67 (0.09)	0.71 (0.09)	t = ‑1.60	0.12	0.48
Positive	0.70 (0.12)	0.74 (0.09)	t = ‑1.32	0.19	0.39
Negative	0.64 (0.10)	0.68 (0.12)	t = ‑1.26	0.22	0.38
**Post-training empathic accuracy score**					
Total	0.71 (0.10)	0.70 (0.10)	t = 0.49	0.63	0.15
Positive	0.73 (0.11)	0.73 (0.12)	t = 0.15	0.88	0.05
Negative	0.69 (0.14)	0.67 (0.13)	t = 0.66	0.51	0.20
**Change in empathic accuracy score**					
**Total**	**0.041 (0.010)**	**‒0.013 (0.094)**	**t = 1.88**	**0.03**	**0.56**
Positive	0.035 (0.105)	‒0.014 (0.093)	t = 1.64	0.05	0.49
Negative	0.045 (0.110)	‒0.012 (0.144)	t = 1.49	0.07	0.45

Repeated measures ANOVA shows that there is a significant group (SCT/control) by time point (pre/post training) interaction (F = 4.254, p = 0.045, η^2^_G_ = 0.02). Post-hoc t-test comparisons of the significant ANOVA effects breaking down task performance show that there were no baseline differences between the Active and Control groups on overall empathic accuracy or on empathic accuracy to positive or negative videos. There was a significant difference on change in empathic accuracy following training, with the Active group showing significantly greater improvement on empathic accuracy compared to the Control group (no further correction for multiple comparisons).

### Subjective Empathy—Interpersonal Reactivity Index

Repeated measures ANOVA shows that there was no group by time point interaction for any of the four subscales of the IRI [F(3,123) = 0.124, p = 0.946]. However, there were significant differences at baseline between the groups on some of the IRI subscales, as is evident from a group by subscale effect on the baseline data [F(3,123) = 2.853, p = .040]. Specifically, there was a significant difference at baseline between the two groups for overall interpersonal reactivity index ratings [t(40) = ‑3.42, p = 0.001]. The CON group showed a significantly lower score on the personal distress subscale and perspective taking subscale compared to the SCT group, as well as a lower average IRI score overall. The significantly lower personal distress and perspective taking scores were also present at the post-training testing, as well as a significant group difference on the empathic concern subscale. However, there were no significant changes on overall IRI score or within any IRI subscale in the CON or the ACT group following training and no between-group differences in change following training (**Table 3**). There were no significant correlations between IRI subscales and EA score at baseline or at follow-up.

**Table 3:** Interpersonal reactivity index score.

	Active N = 24	Control N = 21	Group difference		
			Statistic	*p*-value	Effect size (d)
**Pre-training IRI scores**					
Fantasy	19.38 (4.83)	17.26 (6.17)	t = 1.224	p = 0.229	0.39
Empathic Concern	21.58 (3.56)	19.63 (3.93)	t = 1.684	p = 0.101	0.52
Perspective Taking	23.21 (4.17)	19.79 (4.37)	t = 2.60	**p = 0.013**	**0.80**
Personal distress	14.29 (6.00)	8.11 (4.31)	t = 3.930	**p < 0.001**	**1.16**
**Post-training IRI scores**					
Fantasy	20.29 (5.13)	17.74 (6.82)	t = 1.664	p = 0.105	0.43
Empathic Concern	22.00 (4.04)	19.89 (3.30)	t = 2.219	**p = 0.032**	**0.56**
Perspective Taking	22.83 (4.12)	19.79 (4.87)	t = 2.257	**p = 0.029**	**0.68**
Personal distress	14.79 (6.58)	8.32 (3.42)	t = 4.312	**p < 0.001**	**1.19**
**Change**					
Fantasy	0.92(4.34)	0.47(2.55)	t = 0.417	p = 0.679	0.12
Empathic Concern	0.42(3.54)	0.26(2.42)	t = 0.168	p = 0.867	0.05
Perspective Taking	‒0.38(3.73)	0.00(2.60)	t = ‑0.388	p = 0.700	0.11
Personal distress	0.50(3.78)	0.21(3.36)	t = 0.266	p = 0.792	0.08

Scores on the Intrinsic Reactivity Index subscales. Participants rate each of seven items on each subscales from “Does Not Describe Me Well” (1) to “Describes Me Very Well” (2); total scores can range from 7 to 28. There was a significant difference (bold) on change self-reported perspective taking and personal distress between groups at baseline but there were no significant changes over the course of training in either group and no group differences in IRI subscale score changes.

### Effects of Motivation (IMI) on Training Results

IMI scores were included in a hierarchical linear regression model to examine whether motivation influenced the effect of training. The hierarchical regression confirmed that at stage one, group predicted overall change in EA score [F(1,43) = 3.49, p = 0.034] and accounted for 7.3% of the variance in EA score change. Introducing IMI score explained an additional 22.4% of the variance and this change in R^2^ was significant [F(2,42) = 6.49, p = 0.004]. The linear regression including both group and IMI score demonstrates a significant IMI by group interaction on change in empathic accuracy performance from pre- to post-training [F(1,41) = 10.886, p = 0.002]. Follow-up analyses show that individuals in the SCT group demonstrated a significant correlation between IMI and change in empathic accuracy performance, such that higher overall IMI rating was related to greater improvement in EA score [r(22) = 0.614, p = 0.001], whereas individuals in the control condition did not show a relationship between IMI and change in EA score [r(19) = ‑0.254, p = 0.267]. There were no effects of IMI on the IRI for either group or time point.

## Discussion

Individuals who completed a computerized social cognition training program demonstrated improved performance on a rater objective measure of empathic accuracy while individuals who completed a computer game control condition did not demonstrate any significant changes in their performance on the empathic accuracy task. Importantly, the changes in empathic accuracy in the active social cognitive training group occurred even though the training paradigm did not directly train individuals on the empathic accuracy task. These performance improvements after social cognition training were not specific to positive or negative valence information and instead demonstrate an increased empathic accuracy overall. Higher motivation ratings, as measured by the IMI, were associated with greater performance improvements in the individuals who received social cognition but not the control training, suggesting greater effects in individuals that self-report greater effort and engagement with the training. On the other hand, there were no significant changes in subjective empathy as measured by the IRI self-report scale, indicating that improvements in objective empathic ability were not accompanied by a subjective perception of increased empathic response.

This study is the first to demonstrate that targeted training in broad social cognition may increase empathic abilities, even in healthy individuals with no particular social cognitive deficits. Empathic accuracy, as objectively measured by the correspondence between self-reported affect and observer reported affect, has been argued to be a more ecologically valid measure of emotion recognition that requires real-time integration of visual, auditory and linguistic information (34). This measure may be a more sensitive measure for detection of change in the skills underlying empathy, such as emotion recognition, that constitute the more cognitive components of empathy. Thus, it is unclear whether social cognition training focused around understanding, labeling and reasoning about the affective states of another individual will have an impact on the more affectively generated components of empathy, such as compassion or experience sharing.

These results also demonstrate the importance of measuring motivation to engage in cognitive training protocols. Engagement in the cognitive training protocol has been demonstrated previously to be a necessary component of neuroplasticity focused targeted cognitive therapy, requiring the individual to engage in the targeted behaviors for reasons beyond extrinsically motivated factors such as subject payment (55). Increasing motivation to engage in treatment may be especially relevant for neuroplasticity focused paradigms like social cognitive training (48). Individuals who reported higher levels of motivation on the IMI also tended to show greater behavioral improvements on the empathic accuracy task. However, as motivation was measured at the end of training the directionality of this effect is unclear and this information about individuals who did not complete the training is not available. Further research is necessary to determine the role of motivation in training engagement, the effect of perceived benefit on self-reported motivation, and methods to most effectively engage individuals in training.

Addressing empathic accuracy *via* social cognition training may be beneficial in a variety of contexts. The demonstrated improvement on empathic accuracy suggests that targeted social cognitive training may be useful for individuals with impairments in cognitive skills required for empathy. The individuals who completed the social cognitive training showed significant improvements on the empathic accuracy task while the individuals in the control condition did not; however, we did not detect significant differences between the two groups following training, suggesting that the most improvement was found in individuals who demonstrated lower performance on the task. This suggests there may be limitations to the utility for social cognitive training in otherwise cognitively healthy adults and demonstrates the need to evaluate similar training in individuals with social cognitive impairments. Developmental effects, such as childhood emotional abuse, can negatively impact an individual’s empathic accuracy and this in turn impacts satisfaction in adult marital relationship (56). Development of interpersonal skills, including empathic accuracy, is also important during adolescence as the social, cognitive and biological processes that develop at this time directly influence social cognitive skills and peer relationships (5) and social and emotional adaptation (57). In addition, individuals with psychiatric disorders such as schizophrenia often demonstrate deficits on empathic accuracy (13, 58, 59) and these impairments are associated with poor global social functioning, even after accounting for symptoms and general cognition (17, 59). Conditions and developmental factors, including normal aging, that diminish cognitive resources impact the cognitive more than the affective sharing components of empathy (7). The results of this study suggest that social cognitive training could be effective in clinical samples that are impaired in emotion recognition. Effective interventions could have tremendous benefits since improved ability to accurately infer the thoughts and feelings of others may increase an individual’s ability to make appropriate behavioral responses during ongoing social interactions, leading to better relationship formation and better adjustment outcomes.

Interestingly, despite the objective improvements in the active training group, neither group reported subjective changes in their interpersonal interactions. In studies of empathy, especially those in clinical populations who may show less cognitive insight in general, it is important to assess both subjective and objective measures. As in many domains, there may be a belief-ability gap in which one’s subjective empathy does not as accurately track with performance on objective measures of empathy. Furthermore, individual differences in personality and experience may affect the individual’s judgment of their empathic accuracy. For example, trait positive emotion is associated with higher levels of subjective trait and state empathy, but not better performance on the rater objective empathic accuracy task (60). In this particular instance, subjects did not appear to incorporate any changes in their empathic accuracy abilities into their self-concept of their empathic tendencies. Furthermore, there was not a significant relationship between IRI scores and performance on the empathic accuracy task at either baseline nor follow-up, suggesting that these measures are assessing different components of empathy and that the social cognition training may show greater impact on objective measures than subjective measures.

One limitation of the current study is that individuals are instructed to attend to the emotional changes of a stranger in a standardized video, so it may not fully reflect empathic accuracy when spontaneously perceiving the emotions of others, especially close or well-known others, in everyday life. Thus, the results presented here may be a more valid assessment of interactions with individuals who are less familiar. Once people develop a longstanding interpersonal relationship, their empathic accuracy is also informed by past interactions and thus may be less responsive to training. In other words, it is possible that computerized social cognition training, such as the one presented here, is more effective with regards to individuals’ casual social interactions. Accuracy of emotion recognition may also be affected by the mood of the subject or other state-related factors, though this was not measured in the current study. The same videos were presented in a standard order to all individuals, and while there was no evidence of order effects or effects of gender congruence between the target and the participant in the current study, there may be additional individual differences that impact performance on this measure. Furthermore, as noted above, this type of training may more specifically address the cognitive components of empathy and may not be particularly effective at the affective sharing components, thus it is important to consider which aspects of empathy are impaired in a population receiving training. For example, individuals with conduct disorder and/or callous-unemotional traits show greater impairment in affective empathy than in empathic accuracy (61, 62).

As a self-report measure, the IRI may not be as aligned with current models of empathy as it conflates empathy and sympathy, and the empathic concern subscale does not directly assess true sharing of affective states (63). However, the perspective taking scale appears to have a relatively strong overlap with cognitive empathy and thus is a reasonable primary target for a self-report measure of similar empathic abilities to the empathic accuracy task. In addition, the current study did include a measure of target expressiveness, though empathic accuracy deficits have been shown to vary between highly expressive targets and those with less affective expressiveness (41). Another potential drawback is that the empathic accuracy task uses retrospective ratings of emotional valence by the target person, thus they may not fully represent the experience of the target individual in the moment. Finally, post-training measures were completed shortly after the subject finished training, so the duration of improved performance on empathic accuracy is unclear. In addition, it may take time for objective training-related improvements to be noticed and incorporated into an individuals’ self-concept of their empathic traits. As such, training-related changes in theory of mind or empathy may take longer to translate into changes on subjective measures like the IRI. Future research would benefit from additional follow-up at longer intervals following the completion of training in order to assess the development and temporal stability of training effects.

Of note, the current study only assessed behavioral improvements on the empathic accuracy task following social cognitive training. Neural correlates of empathic accuracy performance are found in regions associated with mental state attribution such as the medial prefrontal cortex and superior temporal sulcus as well as sensorimotor regions in the inferior parietal lobule and the dorsal premotor cortex that could reflect experience sharing (37). Future research should investigate which neural systems drive behavioral performance improvement following social cognitive training. In addition, while there were no significant effects of how long individuals took to complete the prescribed training, online computerized training paradigms are by nature relatively unstructured and allow for different approaches in frequency of training. Additional research is necessary to assess the implementation of targeted computerized training and to assess the training effects of dosage, frequency, and internal versus external motivation. Further investigation on the potential to augment social cognitive training with supplements or hormones like oxytocin (64) would also be beneficial. Finally, as feedback on empathic accuracy has been shown to enhance training of new therapists (65), future research should explore a variety of contexts in which social cognitive training can aid personal, occupational, and educational goals.

In summary, this study is the first to demonstrate that a novel computerized targeted social cognitive training paradigm improved performance on an objective measure of empathic accuracy. While the social cognitive training engages many skills relevant to empathy, it does not directly train empathic accuracy, demonstrating an extension to social cognitive skills beyond those included in the training exercised. Furthermore, individuals who reported higher levels of intrinsic motivation and engagement during the training also showed the largest performance improvements. However, while individuals who received the training showed improved performance, they did not demonstrate changes in subjective assessment of their empathic reactions. This study provides preliminary support for the use of this social cognitive training program to improve skills required for social interactions.

## Data Availability Statement

The datasets generated for this study are available on request to the corresponding author.

## Ethics Statement

The studies involving human participants were reviewed and approved by Harvard University Institutional Review Board. The patients/participants provided their written informed consent to participate in this study.

## Author Contributions

CH, MN, EG, and DD-F conceived and carried out the study and data collection. CH and MN contributed to the development of the social cognitive training program. KH and CH planned and carried out the primary data analyses and contributed to the interpretation of the results. KH drafted the manuscript and all authors provided critical feedback that helped shape the research, analysis and manuscript.

## Funding

This research was supported by the Commonwealth of Massachusetts, Department of Mental Health, a Brain and Behavior Research Foundation NARSAD Independent Investigator Award (PI: Hooker) and the National Institute of Mental Health (R01 MH105246).

## Conflict of Interest

The authors declare that the research was conducted in the absence of any commercial or financial relationships that could be construed as a potential conflict of interest.
