# Supplementary figures and images for: Change in Objective Measure of Empathic Accuracy Following Social Cognitive Training
Source: Front Psychiatry. 2019 Dec 10;10:894. doi: 10.3389/fpsyt.2019.00894 (PMC6914938; doi:10.3389/fpsyt.2019.00894)

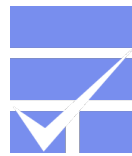

# CONSORT

TRANSPARENT REPORTING of TRIALS

## CONSORT 2010 Flow Diagram

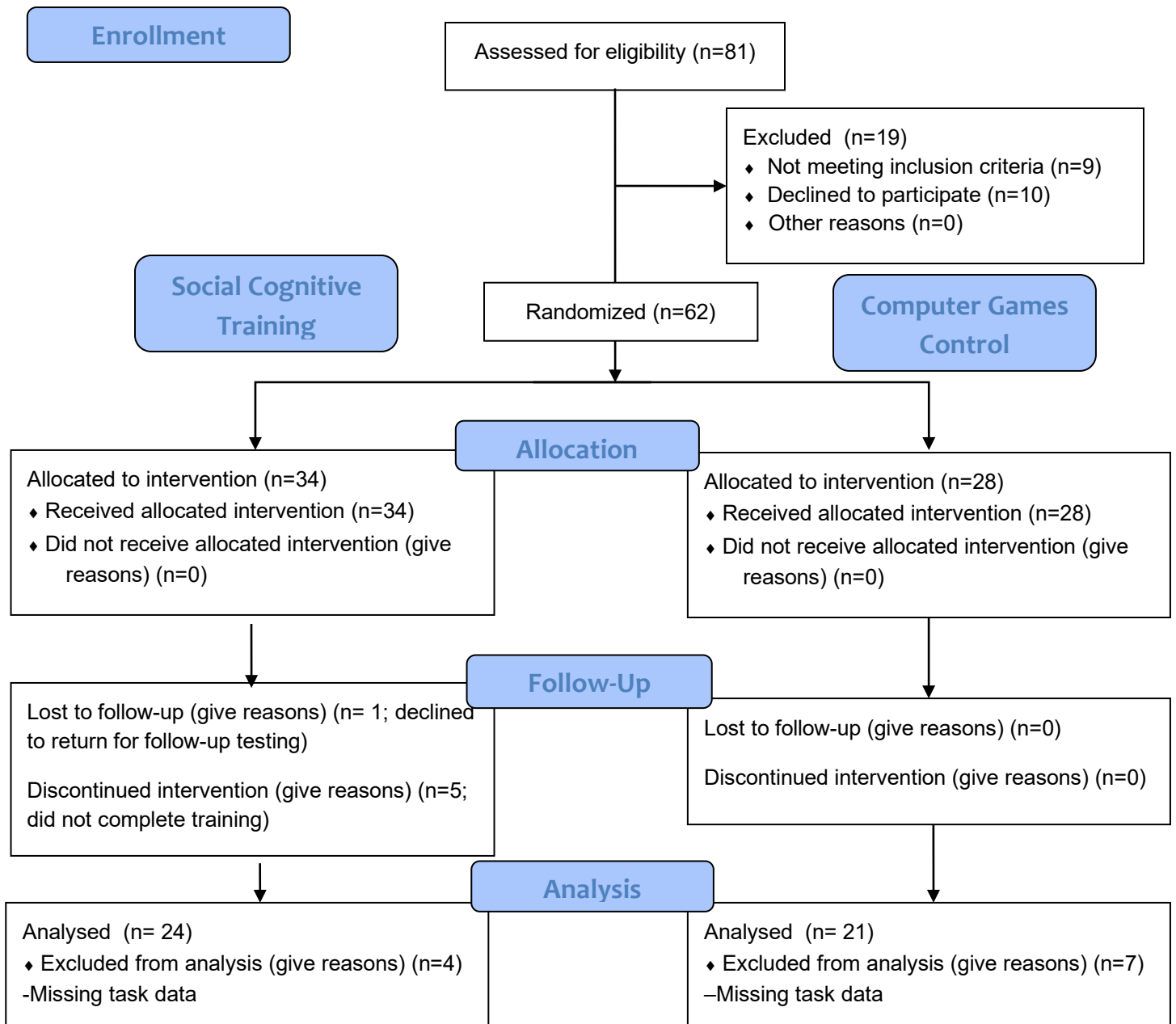

Supplement: Supplementary file 1 [file Image_1.pdf]
