# Supplementary material for: Change in Objective Measure of Empathic Accuracy Following Social Cognitive Training
Source: Front Psychiatry. 2019 Dec 10;10:894. doi: 10.3389/fpsyt.2019.00894 (PMC6914938; doi:10.3389/fpsyt.2019.00894)
Supplement: Supplementary file 2 [file Table_1.pdf]

SOC Study Exercise Descriptions

Exercise Name	SC Domain Trained	Number of Levels	Description
Name that Feeling	Affect Perception	16	Select the label which correctly describes the facial expression of the rapidly presented target face (still images)
Emotion Motion	Affect Perception	17	Select the label which correctly describes the facial expression of the target face (video clip)
Voice Choice	Affect Perception	16	Select the label which correctly describes the target vocal affect (voice prosody)
Second that Emotion	Affect Perception	16	A memory game for facial expression: match pairs of cards that express the same facial affect
Second that Intonation	Affect Perception	17	A memory game for vocal expression: match pairs of cards that express the same vocal affect (prosody)
Match that Feeling	Affect Perception	20	Select the face whose expression matches that of the rapidly presented target face
Face It: Flashback	Social Cue Perception	16	Correctly memorize an increasingly longer sequence of faces
Recognition	Social Cue Perception	17	Select the target face from an array of faces presented from various angles.
Face Facts	Social Cue Perception	13	Memorize visually presented social facts about individuals presented serially
Say What?	Theory of Mind	13	Decide how would a person respond in a given situation (audio scene)
What Happened?	Theory of Mind	16	Decide what most likely happened given the reaction in the clip
Social Scenes	Theory of Mind	17	Rate the likelihood of people's reactions and feelings in certain social situations
